# Deep learning identifies cardiac coupling between mother and fetus during gestation

**DOI:** 10.3389/fcvm.2022.926965

**Published:** 2022-07-29

**Authors:** Mohanad Alkhodari, Namareq Widatalla, Maisam Wahbah, Raghad Al Sakaji, Kiyoe Funamoto, Anita Krishnan, Yoshitaka Kimura, Ahsan H. Khandoker

**Affiliations:** ^1^Department of Biomedical Engineering, Healthcare Engineering Innovation Center, Khalifa University, Abu Dhabi, United Arab Emirates; ^2^Graduate School of Biomedical Engineering, Tohoku University, Sendai, Japan; ^3^Division of Cardiology, Children's National Hospital, Washington, DC, United States; ^4^Department of Maternal and Child Health Care Medical Science, Tohoku University Graduate School of Medicine, Sendai, Japan

**Keywords:** fetal cardiology, maternal-fetal coupling, phase coherence, electrocardiography, deep learning

## Abstract

In the last two decades, stillbirth has caused around 2 million fetal deaths worldwide. Although current ultrasound tools are reliably used for the assessment of fetal growth during pregnancy, it still raises safety issues on the fetus, requires skilled providers, and has economic concerns in less developed countries. Here, we propose deep coherence, a novel artificial intelligence (AI) approach that relies on 1 min non-invasive electrocardiography (ECG) to explain the association between maternal and fetal heartbeats during pregnancy. We validated the performance of this approach using a trained deep learning tool on a total of 941 one minute maternal-fetal R-peaks segments collected from 172 pregnant women (20–40 weeks). The high accuracy achieved by the tool (90%) in identifying coupling scenarios demonstrated the potential of using AI as a monitoring tool for frequent evaluation of fetal development. The interpretability of deep learning was significant in explaining synchronization mechanisms between the maternal and fetal heartbeats. This study could potentially pave the way toward the integration of automated deep learning tools in clinical practice to provide timely and continuous fetal monitoring while reducing triage, side-effects, and costs associated with current clinical devices.

## 1. Introduction

Assessment of fetal wellbeing during pregnancy is of a high importance in ensuring the delivery of a healthy offspring. fetal health monitoring during pregnancy can help in reducing complications associated with asphyxia-mediated damages and in-utero deaths, as it assures regular physiological and psychological development of the fetus throughout trimesters and safe delivery of the mother (1). Globally, it is estimated that more than 2 million babies were stillborn at 28 weeks or more of gestation with a rate of 13.9 stillbirths in every 1,000 births (2). In addition, around 295,000 women died during or after giving a childbirth, where the majority of these deaths (94%) could have been prevented if resources were available (3).

The neurological system of the fetal develops steadily throughout the first, second, and third trimesters of pregnancy (4). The spinal cord synapses start growing first during week 7, where the electrical activity in the brain begins to take place to support spontaneous movements. By the end of the first trimester and more toward the second trimester, involuntary movements like yawning and sucking and other coordinated movements get more visible in ultrasound as the fetal brain continues to develop on weekly basis. The first glance of vital function controls like heart rate and breathing does not appear until the end of the second trimester with the development of the brainstem. Additionally, the cerebral cortex develops by the third trimester giving support to voluntary actions like thinking and feeling, and it develops even further toward the end of pregnancy with motor skills and senses (5, 6).

Currently, ultrasound-based tools are the most commonly used techniques for fetal growth evaluation, biophysical development assessment, and cardiovascular function monitoring (7). However, despite of its reliability and safety, ultrasound relies on the use of acoustic waves in a form of energy that could potentially cause biological effects on the fetus especially when frequent tests are performed (8). In addition, ultrasound requires expensive equipment, highly-skilled technicians, and strong clinical experience, which could raise economic concerns in less developed countries (9). Alongside ultrasound, fetal phonocardiography (PCG) is another common technique for the evaluation of fetal heart function (10). It relies on the auscultation of fetal heart beat sounds non-invasively using a microphone transducer placed on the mother's abdomen (11–13). Although it allows for long-term fetal heart rate measurements, it still does not provide a clinically complete diagnosis due to its noisy nature, high dependency on transducer placement and data acquisition techniques, and non-linear transmission medium for sound waves (14).

Most recently, fetal heart rate and cardiac rhythm monitoring through non-invasive low-cost electrocardiography (ECG) has been widely studied in literature (15–17) to assess fetal wellbeing during pregnancy. It is considered as a non-invasive signal acquisition technique that requires only proper localization of electrodes on the mother's abdomen (18). Upon accurate separation of maternal and fetal ECG waveforms from the abdominal ECG, maternal and fetal heart rate and heart rate variability information can be calculated through detection of R-peaks sequences. The assessment of fetal neurological development can be carried out by analysis of fetal behavioral states and heart rate variability. Heart rate variability was found to grow with pregnancy weeks (19, 20), and coupling between fetal movement and heart rate variability was found to increase with gestational age (21). It was found that fetal motor activity got affected by maternal skin conductance and heart rates, as skin conductance was regarded as an indicator of a sympathetic activation (22). Additionally, it was found in previous studies (23, 24) that the parasympathetic nerves are not involved in the low frequency component of fetal heart rate variability, as they develop by the 18th gestational week. In addition, it was found that the sympathetic nerves, which develops by the 20th week of pregnancy causes the rapid increase in low frequency power. However, due to maternal influence on fetal heart rate variability, it is important to integrate maternal condition within the assessment of fetal neurological maturation (25).

During fetal cardiac cycle, the inferior vena cava allows the blood to enter the heart of the fetus. This behavior may cause maternal information such as psychological and physiological conditions to influence fetal cardiac rhythms, and thus, affect the development of fetal heart during pregnancy. Many studies have been carried in literature to investigate this mechanism and identify matching characteristics in maternal-fetal cardiac interactions. For example, several studies have reported a strong association between maternal physio-psychological states with fetal heart rates throughout gestation (26–28). An increased maternal stress was found to cause an elevation in the heart rate of the fetus (29, 30), whereas a decreased heart rate was found in synchrony with a decreased maternal heart rate during nocturnal activities (31, 32). In addition, maternal exercise that results in an overall increase in maternal heart rate causes hypoxia in the fetus (33). Maternal respiration was found to cause alterations in the synchronization (34, 35), as fast breathing induces the maternal-fetal coupling, while reduced breathing results in higher vagal tone and beat-to-beat differences that reduce this coupling phenomena. Therefore, it is hypothesized that the oscillatory rhythm of maternal respiration is the driving force behind these interactions and the maternal cardiac system affects directly fetal heart rhythms with an acoustic stimulus effect. Moreover, it was found that maternal sleep positioning and patterns significantly impact fetal heart rate and cardiac cycle (32, 36, 37). This correlation between maternal and fetal heart rhythms strongly suggests the possibility of having a hidden maternal-fetal coupling mechanism between their cardiac systems (34, 38), as it is found that fetal suprachiasmatic nucleus could play a pivotal role in transferring maternal cardiac information to the heart of the fetus (39). However, many specific mechanisms leading to this coupling are still unexplained and require further quantification using advanced signal and data analysis techniques.

Conventional analysis techniques for the maternal-fetal cardiac coupling through ECG highlighted the use of phase locking (34), partial directed coherence (PDC) (27), transfer entropy (26, 40), additive auto-regressive processes (41), partial rank correlation (42), and bi-variate phase-rectified averaging (43). An epoch of synchronization was observed in the majority of these studies as a consistent influence of the maternal heartbeats preceding fetal heartbeats. However, there is still a lack of knowledge of an accurate and comprehensive representation of the coupling mechanism, especially with the necessity of applying extra mathematical derivations in current techniques, i.e., phase calculations, to quantify the coupling, which could lead to bias in the observations according to the used method and the type or quality of input data.

In this study, we propose the analysis of cardiac coupling between maternal and fetal heartbeats through deep learning. A deep learning model trained directly on the raw ECG information could potentially explain this relationship without the need of any mathematical derivations or pre-processing steps. To the best of our knowledge, this is the first study to use an artificial intelligence (AI)-based approach to explain the correlation between mother and fetus heartbeats during gestation from a trained machine perspective. AI has been widely used in understanding and resolving many health conditions including cardiovascular-related diseases (44), and deep learning could be a promising approach to reduce the current uncertainties in evaluating the maternal-fetal coupling. We test the reliability of our approach, named deep coherence, by comparing it to a conventional coupling analysis technique, the phase coherence method and its corresponding phase coherence index (λ_*p*_) (45, 46). Our proposed approach ensures several advances relative to previous works. We do not apply any mathematical derivations, pre-processing steps, or signal transformations to the input data. Instead, we let our deep learning model learn freely from the raw ECG information and establish its own learned parameters with regard to coupling scenarios. In addition, we extend the benefits of using deep learning by interpreting these learned parameters to derive the knowledge on coupling decisions in a form of explainable attention heatmaps that mimics the human perception in identifying distinguishable characteristics. Lastly, we minimize the duration needed for ECG recordings to 1 min only, which could potentially reduce the bulkiness of the clinical data acquisition protocols and heavily diminish the need for huge computational demands.

## 2. Results

### 2.1. Preparation of input data

**Figure 1:**
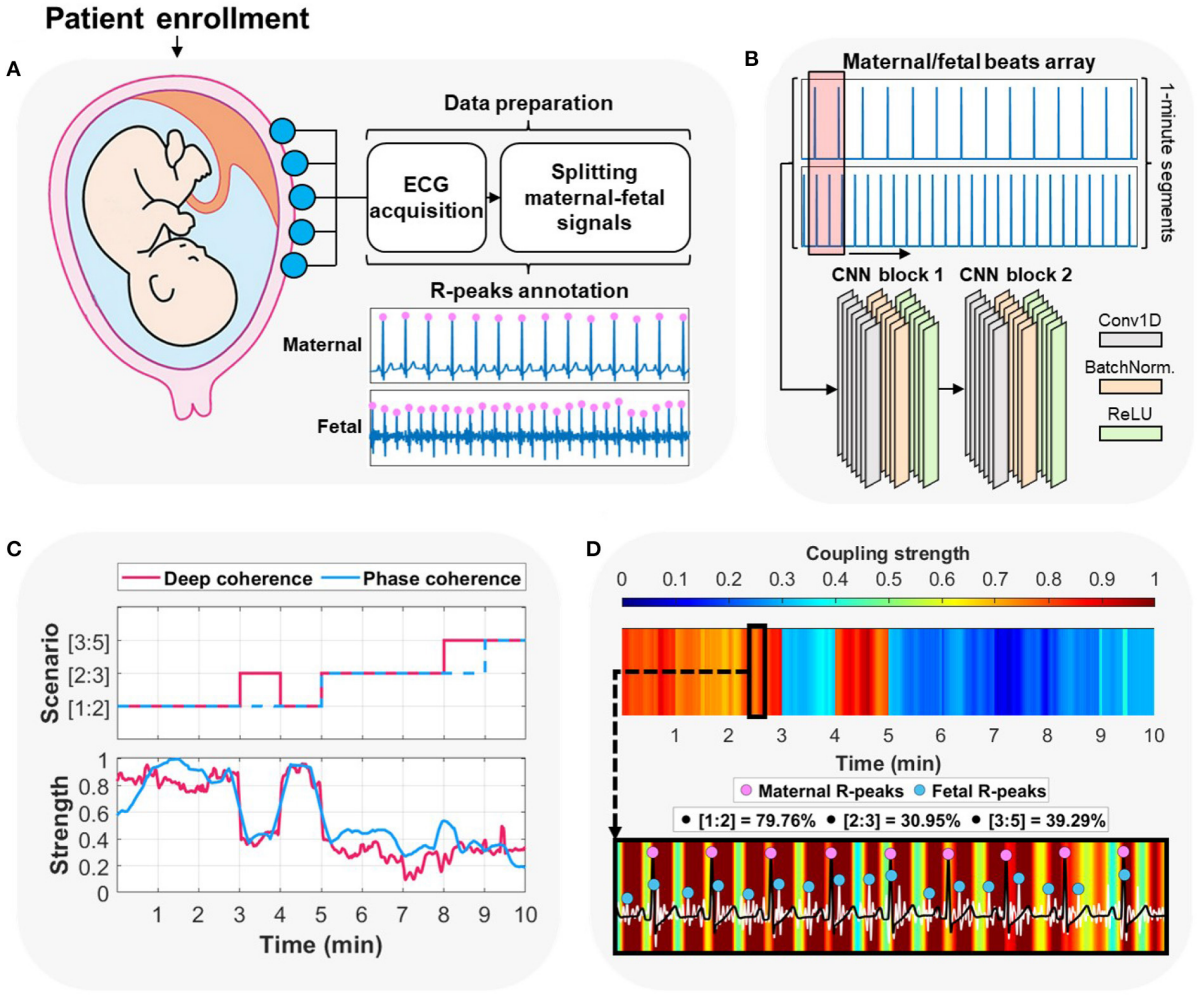
Overview of the proposed approach for maternal-fetal cardiac coupling analysis using deep learning. **(A)** The procedure started by acquiring abdominal electrocardiography (ECG) recording from the pregnant patient enrolled at the hospital/clinic. The method proceeded by splitting the contaminated maternal-fetal signals and detecting their corresponding R-peaks locations. **(B)** The second step was arranging the input to per-minute segments of the maternal/fetal combined heartbeats array and feeding it to a two-block convolutional neural network (CNN). **(C)** The deep learning model was trained on predicting per-minute cardiac coupling scenarios ([1:2], [2:3], or [3:5]). In addition, it allowed for extracting attention to such coupling in a form of a continuous signal using the gradient-weighted class activation mapping (Grad-CAM) technique. We compared deep learning predictions and attentions vs. the ground-truth label (assigned using phase-occurrence counting) and its corresponding phase coherence strength (λ_*p*_) (see Section 4). **(D)** Deep learning allows for explaining the decisions by interpreting an overall attention heatmap image which shows the variations of cardiac coupling strength with time. A zoomed-in heatmap allows for observing patterns between maternal and fetal ECG signals alongside their corresponding R-peaks. We show the original prevalence (%) of each coupling scenario in the 2–3 minutes time interval (deep learning prediction was [1:2]).

Our approach (Figure 1A) starts with minimal data preparation before proceeding with further deep learning analysis. After acquiring abdominal ECG recordings from the enrolled patient, we split the contaminated maternal and fetal signals. Then, we divided each signal (if more than 5 min in length) and its corresponding annotations into shorter 1 min segments (will be merged back at a later stage). To identify the prevalence (in percentage) of various coupling scenarios, we relied on phase-occurrence calculations (see Section 5) between maternal and fetal R-peaks annotations. Such technique is simply an R-peak counting approach based on the various coupling scenarios. Accordingly, we assigned a ground-truth label for each maternal-fetal segment, which was the most prevalent coupling scenario in the segment. The majority (>87%) of assigned coupling labels corresponded to ratios of [1:2], [2:3], and [3:5], therefore, we have selected their corresponding signals as the only data used for analysis in this study. We then split the input data as training set (*n* = 721 segments) and completely-hidden local testing set (*n* = 152 segments) for deep learning. In addition, we used maternal-fetal ECG data from PhysioNet databases (*n* = 68 segments) for extra validation (see Section 5).

### 2.2. Patient characteristics analysis

After segmenting the original patient data and assigning proper ground-truth labels, we have statistically analyzed three demographical information with each coupling scenario in the training and the local testing sets (Table 1). Patients' age, gestational age, and body mass index (BMI) information were statistically analyzed using a one-way analysis of variance (ANOVA) test, where a significant different was observed if the *p*-value was less than 0.05.

**Table 1:** Demographical information of all coupling samples included in the study.

**Variables**	**Training set (Total samples = 721)**				**Testing set (Total samples = 152)**				**Combined (Total samples = 873)**			
	**[1:2]**	**[2:3]**	**[3:5]**	*p* **-value**	**[1:2]**	**[2:3]**	**[3:5]**	*p* **-value**	**[1:2]**	**[2:3]**	**[3:5]**	*p* **-value**
Samples (Total mothers)	446 (59 mothers)	141 (24 mothers)	134 (31 mothers)		73 (21 mothers)	42 (14 mothers)	37 (15 mothers)		519 (80 mothers)	183 (38 mothers)	171 (46 mothers)	
Gestational age (weeks)	28.50^*^ (25.45–34.16)	33.20^*^^‡^ (29.66–36.12)	27.60^‡^ (25.13–34.24)	<0.001	30.00^*^ (26.08–34.46)	34.55^*^^‡^ (29.80–36.21)	27.20^‡^ (25.16–33.26)	0.010	29.20^*^ (25.59–34.20)	33.20^*^^‡^ (29.70–36.13)	27.20^‡^ (25.14–34.01)	<0.001
Maternal age (years)	33.00^*^ (28.51–34.80)	26.00^*^^‡^ (24.53–32.86)	30.40^‡^ (29.51–34.68)	<0.001	34.00^*^ (30.78–37.55)	32.00^*^ (27.25–35.65)	32.00 (26.97–36.49)	0.028	33.00^*^ (29.00–35.52)	30.00^*^^‡^ (25.17–33.65)	31^‡^ (28.72–35.27)	<0.001
Maternal BMI (kg/m^2^)	22.34^*^^¶^ (20.83–24.50)	24.53^*^ (22.77–26.02)	26.21^¶^ (23.70–26.65)	<0.001	23.90 (21.69–26.62)	23.78 (22.23–28.03)	23.07 (21.47–26.72)	0.406	22.34^*^^¶^ (21.02–25.22)	24.53^*^ (22.51–26.76)	24.14^¶^ (22.62–26.78)	<0.001

All values are represented as median (inter-quartile range) or *n* (%).

Bold *p*-value: Significant difference (*p* < 0.050) between the three coupling scenarios.

* Significant difference between [1:2] and [2:3] coupling scenarios.

¶ Significant difference between [1:2] and [3:5] coupling scenarios.

‡ Significant difference between [2:3] and [3:5] coupling scenarios.

BMI, Body mass index.

In the training set, all mothers were below 40 years old. A significant difference (*p*-value < 0.001) was observed in maternal age especially with the [2:3] coupling scenario that had the lowest median of age (26 years old). In contrast, [2:3] coupling samples had higher gestational age inter-quartile range (IQR) of 29.66–36.12 weeks (*p* < 0.001) compared to the other coupling scenarios. It is worth noting that [1:2] and [3:5] had close IQR values in both maternal age and gestational age, thus, they had no significant difference observations. In maternal body mass index (BMI), the lowest median values were in the [1:2] coupling scenario (22.34 kg/m^2^) that were statistically significant (*p* < 0.001) from the [2:3] and [3:5] scenarios.

The local testing set had significant differences in gestational age and maternal age only, with *p*-values of 0.010 and 0.028, respectively. Similarly to the training set, [2:3] coupling scenario had the highest median of gestational weeks (34.55 weeks) and [1:2] coupling scenario had the highest IQR in maternal age with 30.78–37.55 years. Moreover, maternal BMI was not found significant between the three coupling scenarios, as they had roughly a median range of 23.78–23.07.

When combining both sets altogether, the three demographical information were found significant. The [2:3] coupling scenario remained the lowest and highest in terms of median maternal age and gestational age, respectively. Similar to the training set, [3:5] coupling had the lowest median gestational age of 27.20, while the highest median maternal age was for the [1:2] coupling scenario with 33.00. In the BMI variable, the [1:2] coupling had the lowest median value, which made it significantly different from both of the other coupling scenarios.

### 2.3. Deep learning prediction of coupling scenarios

We tested the ability to discriminate between the three coupling scenarios by training a complete deep learning model (Figure 1B). The model was trained on extracting adaptive features from the raw R-peaks locations of the maternal and fetal ECGs. We designed the model to be as simple as possible [two convolutional neural networks (CNN) blocks] to reduce the computational demand while at the same time maintain high levels of performance (47) (see Section 5). In addition, CNNs provide stable gradients and allows for a better control over the receptive field size and memory.

**Figure 2:**
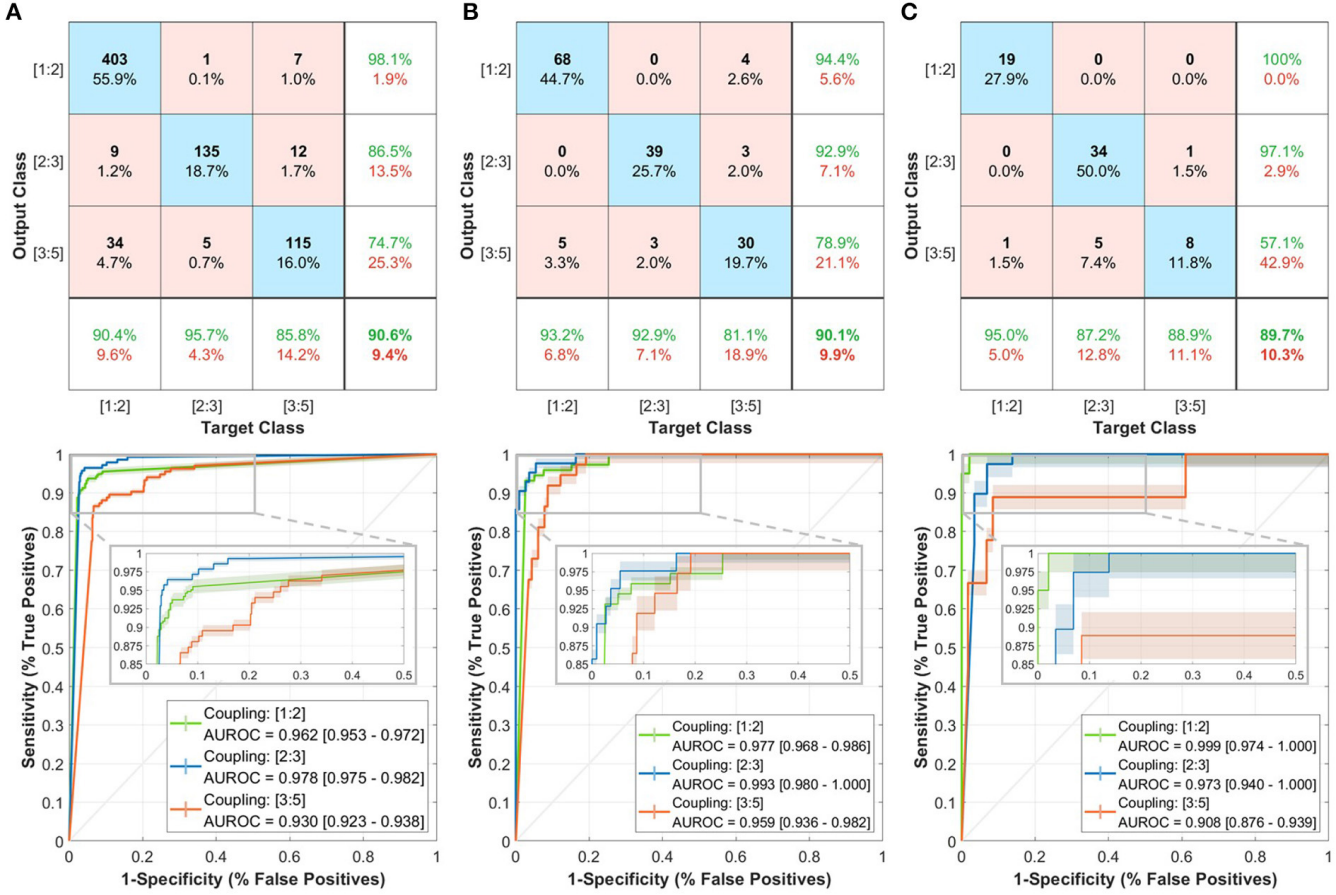
Performance of the trained deep learning model in predicting the three coupling scenarios including confusion matrices (top row) and receiver operating characteristics (ROC) curves (bottom row). **(A)** Training set. **(B)** Local testing set. **(C)** PhysioNet testing set. In the confusion matrix, the bottom row shows the sensitivity, right column shows the precision, and bottom-right corner the overall accuracy. Each of the ROC curves includes a shaded region to represent the 95% confidence interval (CI). A zoomed-in view of the ROC curves shows the interval of more than 50% specificity.

We validated the trained model initially on the training set through a leave-one-out (LOO) cross-validation mechanism (Figure 2A, top row). The model had an overall accuracy of 90.6% in predicting the three coupling scenarios. In addition, the model had high sensitivity in predicting the [1:2] coupling scenario with 95.7%. The lowest precision value was for [3:5] coupling (74.7%) which could be due to similarities found between the [1:2] and [3:5] coupling scenarios from the model's perspective. The area under the receiver operating characteristic (AUROC) was relatively high for the three classes of at least 0.930 (Figure 2A, bottom row). Furthermore, the prediction of the three classes had high confidence with intervals less/more than 0.01.

Then, we tested the model on the completely-hidden local testing set to ensure more validity of the proposed approach. The model achieved a 90.1% accuracy level with close sensitivity and precision measures to the training set predictions (Figure 2B, top row). Moreover, the AUROC had slightly higher values with at least 0.959 (Figure 2B, bottom row).

Lastly, we have evaluated the model on the PhysioNet testing set to check the performance on an external patient cohort (Figure 2C, top row). The accuracy in discriminating the three coupling scenarios was closely similar to that in the training and testing sets (89.7%). The prediction of [1:2] had a 100% precision and 95% sensitivity, which was higher than the other two classes. The AUROC exhibited close performance with at least 0.908 (Figure 2C, bottom row).

### 2.4. Explaining maternal-fetal cardiac coupling

**Figure 3:**
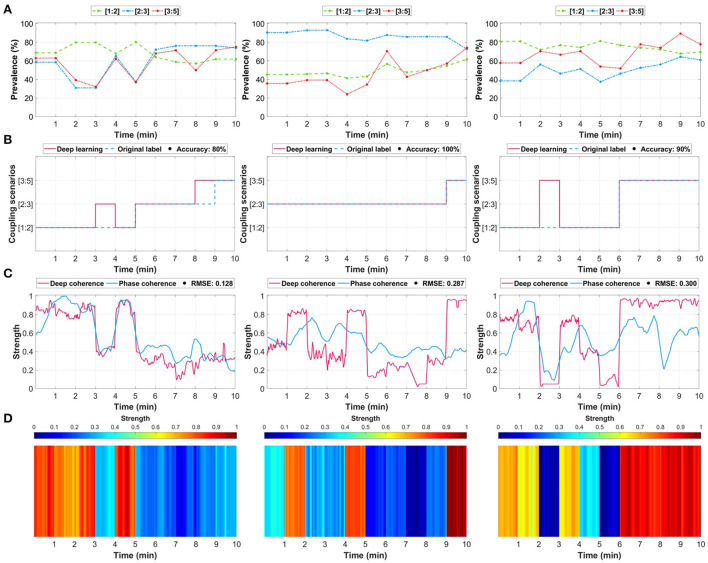
Three examples of maternal-fetal coupling assessment through deep learning relative to the selected ground-truth method (phase-occurrence counting and phase coherence (λ_*p*_)). **(A)** Determining the ground-truth label using the phase-occurrence counting technique (see Section 4). The values indicate the prevalence (%) of each coupling scenario, and the highest was assigned as the ground-truth label. **(B)** The predictions of the trained deep learning model relative to the original label. **(C)** The extracted deep learning attention (deep coherence) using the gradient-weighted class activation mapping (Grad-CAM) technique was considered as a representation of coupling strength relative to the conventional phase coherence (λ_*p*_). **(D)** An illustration of the coupling attention heatmap extracted from the deep learning model.

Training a deep learning model using CNN for the purpose of cardiac coupling prediction allows for extracting additional attention-based information about the decisions, which transforms the regular black-box train-predict mechanism to an explainable and informative approach. We take the advantage of CNN in extracting unique patterns based on the decisions of the network to obtain machine-based understanding of the maternal-fetal cardiac coupling. Therefore, we assessed the three coupling scenarios (Figure 3) to extract deep coherence from the trained model and compared it with the commonly used phase coherence (λ_*p*_) (45, 46).

We first identified the phase occurrence of each coupling scenario (Figure 3A) and assigned a ground-truth label accordingly. Then, we trained the model and compared its predicted coupling scenarios vs. the original assigned label (Figure 3B). We extended the analysis by extracting the attention (deep coherence) to the predicted coupling scenarios for each 1 min maternal-fetal R-peaks segment (Figure 3C) and compared it with the λ_*p*_ metric. The attention was extracted using the gradient-weighted class activation mapping (Grad-CAM) technique (48). Using this technique, the attention shows the most important regions in the input that derived the model's prediction of a certain class, i.e., coupling scenario. Lastly, we illustrated this attention (deep coherence) as an attention heatmap to obtain unique patterns (vertical colored lines) for every predicted coupling scenario (Figure 3D).

Although the model predicted almost all segments correctly, it did not predict few segments due to the close prevalence of the three coupling scenarios. For example, in Figure 3A left column, the model failed to predict minute 4 and minute 9, which have resulted in an accuracy of 80%. However, such missed predictions could be due to the close prevalence of phases in the three coupling scenarios ([1:2]: 68%, [2:3]: 65%, and [3:5]: 62%), which does not necessarily mean that the predictions were totally wrong. Similarly, this was repeated in the third example (Figure 3, right column) at minute 3 between [1:2] and [3:5] coupling ([1:2]: 77% and [3:5]: 67%). The deep coherence was closely following the same pattern of the strength of phase coherence across minutes. The first scenario shown in Figure 3C (left column) had a 0.128 root mean square error (RMSE) although it had two miss-predicted segments. Accordingly, the middle and right column examples had a 0.287 and 0.300 RMSE, respectively.

**Figure 4:**
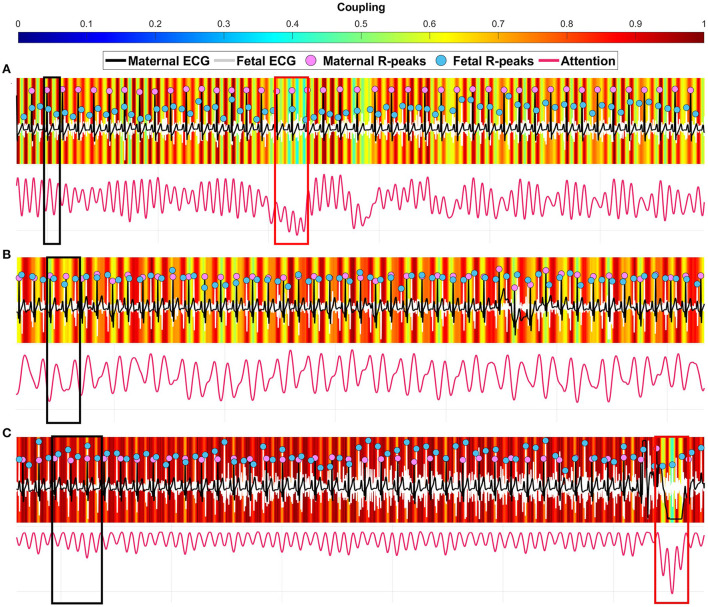
Three examples of coupling heatmaps extracted from the deep learning model using the gradient-weighted class activation mapping (Grad-CAM) technique overlapped with maternal-fetal electrocardiography (ECG) signals. **(A)** correctly predicted [1:2] coupling 30 s segment. **(B)** correctly predicted [2:3] coupling 30 s segment. **(C)** correctly predicted [3:5] coupling 30 s segment. The black boxes show a strong coupling region for each scenario. The red boxes show a weak coupling region.

We extended the analysis by viewing the attention heatmaps for each coupling scenario relative to the original maternal and fetal ECG signals (Figure 4). The attention to the [1:2] coupling scenario was narrower (vertical red lines) than the other two scenarios (Figure 4A). In addition, the [2:3] coupling scenario had wider attention (Figure 4B) which could imply detecting more beats in both signals. In the [3:5] coupling scenario, the heatmap showed an attention that is spread allover the signals with less variability between high and low attentions (Figure 4C). In general, as the number of R-peaks increases, the heatmaps spread wider in terms of attention to those R-peaks locations.

### 2.5. Analysis across the whole dataset

**Figure 5:**
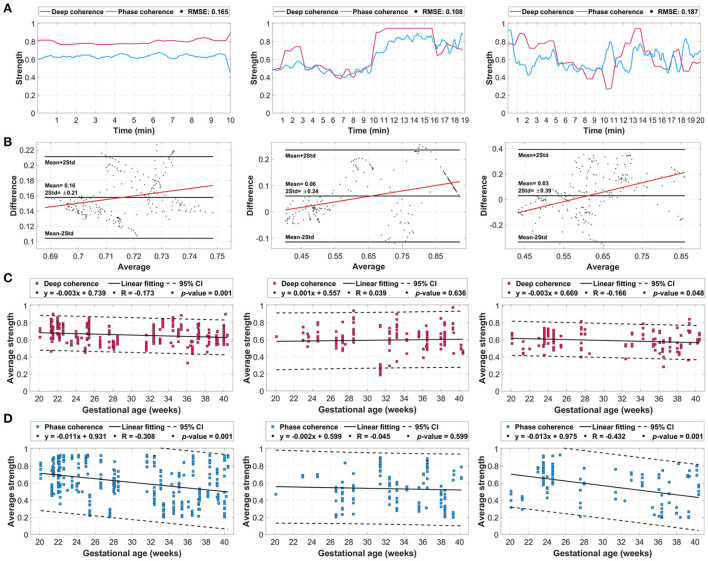
The averaged deep coherence and phase coherence (λ_*p*_) values extracted from the whole dataset with additional evaluation with respect to gestational age. **(A)** The overall deep coherence and phase coherence for coupling scenarios [1:2] (left column), [2:3] (middle column), and [3:5] (right column). **(B)** Bland-Altman plots between the overall deep learning attention and λ_*p*_ signals. **(C)** Deep coherence relative to gestational age in weeks. **(D)** Phase coherence relative to gestational age. The coupling-gestational age plots include the 95% confidence interval (CI) and linearly fitted line characteristics.

The averaged deep coherence and phase coherence across each coupling scenario group showed strong agreements with low levels of error (Figure 5A). For the [1:2] coupling, both methods had a strength of coupling in the range of 0.6 to 0.8 with an RMSE of 0.165. The [2:3] coupling had the lowest error (RMSE: 0.108) with a similar trend going through minutes between deep learning and λ_*p*_. The [3:5] coupling scenario had a 0.187 error with a similar pattern during minutes 4–10 and 13–18. In general, deep coherence was stronger in reflecting the coupling (in terms of amplitude).

The Bland-Altman plots (Figure 5B) explained the changes in the average and difference in coupling between deep learning and λ_*p*_ for each scenario. The [1:2], [2:3], and [3:5] coupling scenarios had mean differences of 0.16±0.21, 0.06±0.24, and 0.03±0.39, respectively. All three scenarios had a linear fitting that is positive (increasing) with respect to the average coupling with the majority of points being within the range of twice of the standard deviation. In addition, there was a bias toward deep learning values due to having a positive mean in all three scenarios, however, [1:2] had the highest positive mean with 0.16.

With respect to gestational age (during 20–40 weeks), both deep coherence and phase coherence had unique patterns that were almost similar for [1:2] and [3:5] coupling scenarios (Figures 5C,D). For [1:2] coupling, deep learning had a decreasing trend throughout gestation (Figure 5C, left column) with a linear fitting correlation (R) of −0.173 and a *p*-value of 0.002. Similarly, λ_*p*_ had a decreasing pattern with gestation (Figure 5D, left column) with an *R*-value of −0.308 and *p*-value of 0.001. In [2:3] coupling, the coupling was increasing as gestation develops (*R* = 0.039) for deep learning (Figure 5C, middle column), and it had the opposite decreasing pattern in λ_*p*_ (*R* = −0.045) (Figure 5D, middle column). In both methods, the fitting was not significant with 0.636 and 0.599, respectively. Lastly, for [3:5] coupling, a significant linear fitting was observed in both deep coherence (Figure 5C, right column) and phase coherence (Figure 5D, right column) with *p*-values of 0.048 and 0.001, respectively. The coupling was decreasing in both methods with respect to gestational age with *R*-values of −0.166 and −0.432 for deep learning and λ_*p*_, respectively. It is worth noting that smaller confidence intervals (CI) were observed for deep learning compared with the conventional λ_*p*_ metric.

## 3. Discussion

In this study, we have investigated the reliability of using deep learning in quantifying the synchronization between the mother and fetus cardiac rhythms throughout gestation. To the best of our knowledge, this is the first study to explore the feasibility of utilizing deep learning for this purpose. This comes with the knowledge that fetal cardiac changes could be potentially driven by the maternal heart function. Therefore, deep learning could play a pivotal role in explaining these relationships numerically and visually as the pregnancy progresses. Such analysis could guide the assessment of the fetus from early gestation up to late gestation, and could highly ease the monitoring of any abnormal behaviors as the heart of the fetus continue to grow. Moreover, the integration of ECG-based information alongside the commonly used ultrasound tools would aid clinicians in providing timely and continuous assessment of maternal-fetal cardiac correlation with less triage and risks on the pregnant mother and fetus.

The assessment of fetal heart growth during pregnancy is of a substantial importance in ensuring proper development of their physical and psychological systems. In addition, this should be provided in parallel with proper monitoring of the mother's cardiac changes during gestation, as it has been shown that they highly affect the fetal cardiac system (26, 35). Our study has demonstrated this correlation by effectively distinguishing three coupling scenarios with three different ratios of maternal and fetal heartbeats. Predictions of the pattern of synchronization could be crucial in evaluating the linkage between maternal and fetal hearts throughout gestation, which has been illustrated as variation in the deep learning attention (deep coherence) to the coupling scenario from week 20–40 (Figures 5C,D).

The prevalence of coupling scenarios within each 1 min maternal-fetal R-peaks segment had high impact on the overall accuracy of the trained model. Although the trained model had almost 90% accuracy in predicting all sets, it had few miss-predicted segments that showed close prevalence percentages (almost 5%) between the three coupling scenarios (Figure 3B). Where it was considered as a wrong prediction relative to the assigned ground-truth label, it could be a correct prediction from the machine perspective. This comes with the knowledge that the model was trained on high number of samples to best differentiate between the three scenarios and to optimally select the coupling representation within each segment. Additionally, it could be less accurate to call a coupling representation only by the highest count of maternal-fetal heartbeats, especially with close prevalence values. In contrast, this was done in deep learning through extraction of deeper features from both maternal and fetal R-peak signals and after learning (during training) from the information of the whole training set.

Our proposed deep coherence and its corresponding Grad-CAM attention heatmaps could provide a more thorough interpretation of the relationships between mother and fetus heartbeats. This explanation mechanism of deep learning decisions suggests unique patterns (as vertical colored lines) of each coupling scenario that can be visualized for each patient. This advantage is lacking from conventional techniques, i.e., phase coherence, thus, it allows for extending current assessment applications of fetal wellbeing throughout gestation with direct overlap on the original ECG signals. For example, we have observed patterns that include thinner important regions (high coupling—red color) in the [1:2] coupling scenario and get spread wider while progressing to [2:3] and [3:5] coupling scenarios (Figure 4). This could be illustrated as a one-to-one relationship in [1:2] coupling which resulted in thin red colors, while wide regions indicated the contribution of more parts within the segment, i.e, more R-peaks. The ability to visualize such difference through deep learning could potentially facilitate new protocols in the evaluation of maternal-fetal synchronization, especially in the presence of cardiovascular diseases. Although other conventional techniques are highly efficient in characterizing the coupling, deep learning could provide more generalizability through learning patterns from big data, Therefore, it could be a promising data-driven approach that is comparable to conventional mathematical techniques. In this study, we propose deep learning for the first time for the purpose of maternal-fetal coupling analysis, however, more testing under unusual cardiac situations should be evaluated.

With regard to coupling behavior as the pregnancy develops, our findings were close to what have been reported in the literature (26, 49). Dividing the gestational age into two periods less than or more than 30 weeks provides better analysis of the attention and λ_*p*_ with respect to fetal heart growth. In our findings (Figures 5C,D), both methods showed a drop in the [1:2] coupling scenario going from the first to the second gestational period, which is an evidence on proper fetal cardiac development that reaches higher heart rates than the maternal (50). Additionally, the [2:3] coupling scenario with deep learning attention had an increasing pattern with gestational age as an indication of more fetal heart activity closer to late pregnancy, which matches our previous findings (49). Interestingly, the [3:5] coupling scenario had a significantly falling linear fit from 20 weeks up to 40 weeks. Such behavior could illustrate reductions in the fetal heart rhythms as pregnancy develops if they were high at the early gestational weeks, which might be an approach to stabilize the heart function of the fetal in late gestation.

Although our study showed a potential in explaining maternal-fetal synchronization using deep learning, we would like to highlight some limitations. We demonstrated the potential of deep learning in discriminating between three coupling scenarios, however, the dataset we used did not include patients with maternal or fetal cardiac abnormalities. Thus, it would be essential to explore the impact of heart diseases on the model's performance. In addition, our study showed significant increasing or decreasing patterns of coupling scenarios with respect to gestational age. A follow-up study of the same patient from early to late pregnancy would boost the findings on case-by-case analysis and could elaborate more on patient-specific coupling mechanisms throughout gestation. Furthermore, due to the limited knowledge in the field and the lack of clinical data in the used dataset, the coupling is still unclear relative to fetal behavioral states. Since fetal activity affects fetal heart rate, it is expected that such activity can influence the coupling. Therefore, the inclusion of fetal behavioral states within the deep learning model may strengthen the predictive ability during the assessment of fetal neurological development.

Future work for this study would consider the inclusion of larger sets of patients with emphasis on cardiovascular diseased pregnant women. Moreover, a complete patient-by-patient-based study throughout gestation would enhance the understanding of coupling progression and fetal growth. Lastly, our deep learning model could be expanded to include more layers, which results in increased levels of learning from larger input data.

## 4. Conclusion

Our study revealed detailed information on the coupling between the mother and the fetus during gestation from a trained machine perspective. Using a simple deep learning model, we were able to effectively and significantly explain the synchronization mechanism in three major beat-to-beat coupling scenarios ([1:2], [2:3], and [3:5]). Our study paves the way toward the integration of deep learning tools in clinical settings alongside current ultrasound devices to ensure timely and continuous fetal growth monitoring while reducing any triage or side-effects on the mother or her fetus.

## 5. Methods

### 5.1. Dataset

#### 5.1.1. Training and local testing sets

The training and local testing sets used in this study included a total of 109 pregnant women (training: 74, testing: 35) recruited at the Children's National Hospital, Washington, United States (15 patients, 13.8%), Tohoku University Hospital, Sendai, Japan (77 patients, 70.6%), and Kanagawa Children's Medical Center, Kanagawa, Japan (17 patients, 15.6%). All women had no cardiovascular disorders and with healthy fetal cardiac conditions. The acquisition protocol of abdominal ECG signals was approved by Children's National Hospital Institutional Review Board (IRB) and Tohoku University (IRB: 2015-2-80-1 and 2020-1-951) with appropriate institutional agreements. A written consent form was obtained from all patients before participating in any data acquisitions. All experiments were performed in accordance with relevant guidelines and regulations of the Declaration of Helsinki.

Patients' age was ranging between 20 and 45 years old and had a gestational age range of 20–40 weeks. A data acquisition device for abdominal ECG monitoring was used to bi-polar record the signals from 12 electrodes placed on the mother's abdomen. The obtained signals were sampled at 1 kHz with a 16-bit resolution. Signals length for each participant was variable but all were recorded for at least 1 min and at most 20 min. All recordings were taken while the participant was lying in a supine position.

Each abdominal ECG recording is a composite of maternal signal, fetal signal, and noise. To separate these components, the recording was filtered using a band-pass filer with a bandwidth of 0.05–100 Hz and a notch filter to remove any powerline interference noises (50/60 Hz) caused by electronic devices. The fetal ECG signal was then separated from the composite recording by employing a cancellation technique of the maternal ECG signal as well as the technique of blind source separation with reference (BSSR) (51, 52). After obtaining the extracted fetal ECG signal, R-peak locations were detected by a custom-made MATLAB routine program (49, 53).

#### 5.1.2. PhysioNet testing set

PhysioNet testing sets were obtained from the abdominal and direct fetal electrocardiogram (ADFE) database (54) and the PhysioNet/Computing in Cardiology (CinC) 2013 challenge (55).

The ADFE set included multi-channel ECG signals recorded from five pregnant women (38–41 weeks of gestation) at the Department of Obstetrics, Medical University of Silesia, Katowice, Poland. All signals were recorded using a KOMPOREL system (ITAM Institute, Zabrze, Poland) of four electrodes located around the navel alongside a reference spiral electrode for direct scalp fetal ECG recording. Signals (5 min long) were sampled at a rate of 1 kHz and a resolution of 16-bit. All R-peaks locations were annotated from the direct fetal ECG signal using the KOMPOREL system and verified at a later stage by a group of cardiologists.

On the other hand, PhysioNet/CinC challenge set included 75 abdominal ECG recordings (training set 'a' and extended 'a') obtained from multiple resources. All signals were recorded for 1 min and sampled with 1 kHz sampling rate. The R-peaks annotations were provided as part of the challenge, as they were acquired from a direct scalp fetal ECG signal.

In both sets, fetal R-peaks annotations were provided, however, maternal R-peak locations were missing. Therefore, we performed R-peaks detection on all abdominal ECG signals considering that the maternal R-peaks are stronger in amplitude and easily detectable from the signals. The detection was performed using the famous Pan-Tompkins algorithm in MATLAB (56).

### 5.2. Segmentation and ground-truth labeling

All ECG signals (maternal and fetal) alongside their corresponding R-peaks annotations were initially segmented into 1 min segments. This resulted in a total of 802 segments in the training set, 175 segments in the local testing set, and 78 segments in the PhysioNet testing set. In each segment, prevalence percentages of different coupling scenarios between maternal and fetal R-peaks annotations were determined through a phase-occurrence calculations approach (57).

In this approach, we calculated the occurrence of coupling ratios relative to maternal heartbeats. Accordingly, ratios were calculated by first counting the number of fetal beats occurring per one, two, and three maternal beats intervals. A one maternal beat was considered to be the interval located between the first maternal R-peak and the second maternal R-peak. Two maternal beats were considered to be the interval between the first R-peak and the third R-peak. Three maternal beats were considered as the interval from the first R-peak and fourth R-peak. Fetal beats were counted if they were located within such intervals. After counting all available maternal-fetal beats per interval (coupling count), the prevalence ratio (%) of each was calculated by dividing the number of occurrence of a particular coupling count by the total number of occurrences of all the other occurring coupling counts.

To label segments with a ground-truth coupling scenario, the highest coupling prevalence ratio among other occurring ratios was selected as an overall representation of the coupling in the selected segment. After assigning ground-truth labels, the majority (>87%) of coupling scenarios were for ratios [1:2], [2:3], and [3:5], therefore, they were the only selected scenarios used for any further analysis. This have resulted in 721, 152, and 68 segments in the training, local testing, and PhysioNet testing sets, respectively, after discarding segments with any other coupling scenarios.

### 5.3. Deep learning modeling

#### 5.3.1. Model architecture

We arranged the input segments of maternal and fetal heartbeats as peaks-only signals (Figure 1B), where each R-peak location had a value of 1 on a 1 min segment of zeros (60,000 samples, vector: [2, 60,000]). Then, we designed the structure of the deep learning model to be based on a simple two-block CNN (Figure 1). A simple CNN model does not require high computational and memory demands, especially in clinical practice. Each CNN block consisted of a one-dimensional (1D) convolutional layer, a batch-normalization layer, and rectified linear unit (ReLu) layer. The first layer had a convolutional kernel size of [2, 1,024] to extract 8 filters' features at each step (stride: [1, 1]) from both maternal and fetal input signals at once. In addition, the second layer had a smaller convolutional kernel size of [2, 512] with 12 filters and stride of [1, 1]. To handle any data bias issues caused by the unbalanced labels, we included a fully-connected layer with soft-max and weight-modified classification layers that assigned class weights empirically.

#### 5.3.2. Training and prediction

The proposed model was trained using the adaptive moment estimation (ADAM) optimizer with a mini-batch size of 12 and a maximum of 15 epochs. The learning rate was assigned as 0.001 with an L2-regularization of 0.0001. On each epoch, the mini-batch was shuffled randomly to reduce the bias during training.

We validated the proposed model first using a leave-one-out (LOO) cross-validation scheme (58, 59). A total of 721 iterations were followed (number of training data samples), where on each iteration, an *i*th subject was used for testing and the remaining *n*–1 subjects were used for training until the whole set is evaluated. Then, we tested our trained model on a completely-hidden local testing set as described earlier (152 samples). For more validation, we tested the trained model on the PhysioNet testing set (68 samples) and observed the performance.

### 5.4. Phase coherence index

We compared our deep learning coupling attention with a commonly used mathematical approach based on phase coherence index (λ_*p*_) (45, 46). At any selected oscillatory cycle between the two signals (maternal and fetal R-peaks), the instantaneous phase (φ(*t*_*k*_)) was calculated as,


(1) $$\begin{array}{l} \varphi \left(t_{k}\right)=\frac{2\pi \left(t-t_{k}\right)}{\left(t_{k+m}-t_{k}\right)}+2\pi k \end{array}$$

where *t* and *t*_*k*_ are the timings of the selected fetal and maternal R-peaks, respectively, and *m* is the total number of maternal heartbeats in each coupling scenario, i.e., 1, 2, or 3.

Then, the relative phase of occurrence (Ψ(*t*_*k*_)) of the maternal R-peaks with respect to fetal R-peaks was calculated as,


(2) $$\begin{array}{l} \text{Ψ}\left(t_{k}\right)=\frac{\varphi \left(t_{k}\right)\;\mathrm{mod}\;2\pi }{2\pi } \end{array}$$

In the case of a strong synchronization between both signals, a syncrogram with parallel horizontal lines is usually observed, whereas no synchronization shows a randomly distributed points on the syncrogram.

Lastly, an estimation of the phase coherence index (λ_*p*_(*t*_*k*_)) was provided in the range of 0 to 1 by quantifying the relative phase (Ψ(*t*_*k*_)) in the selected time window (*t*_*w*_) as,


(3) $$\begin{array}{l} \lambda_{p}\left(t_{k}\right)={\left\|\frac{1}{N}\overset{k+N/2}{\underset{j=k-N/2}{\sum }}e^{i\text{Ψ}\left(t_{j}\right)}\right\|}^{2} \end{array}$$

where *N* is the number of expected R-peaks within the selected time window *t*_*k*_ − *t*_*w*_/2 ≤ *t*_*j*_ < *t*_*k*_ + *t*_*w*_/2. In this study, *N* was selected as 15 due to having shorter signals (1 min segments).

## Data availability statement

Local patient data used in this study will be provided by contacting the corresponding author upon a reasonable request. Requests to access the main dataset should be directed to mohanad.alkhodari@ku.ac.ae or ahsan.khandoker@ku.ac.ae. All codes for data preparation, deep learning trained model, and post-classification analysis using deep coherence and phase coherence are available at: https://github.com/malkhodari/Alkhodari_frontiers_pediatrics.git.

## Ethics statement

The studies involving human participants were reviewed and approved by The acquisition protocol of abdominal ECG signals was approved by Children's National Hospital Institutional Review Board (IRB) and Tohoku University (IRB: 2015-2-80-1 and 2020-1-951) with appropriate institutional agreements. A written consent form was obtained from all patients before participating in any data acquisitions. All experiments were performed in accordance with relevant guidelines and regulations of the Declaration of Helsinki. The patients/participants provided their written informed consent to participate in this study.

## Author contributions

MA, NW, and AHK: designed the research idea. MA: performed the literature search, algorithm implementation, statistical analysis, deep learning modeling, and wrote the initial draft of the manuscript. AK and YK: acquired data. NW, MW, RA, and KF: prepared the data. YK and AHK: supervised the research. MA, NW, MW, RA, KF, AK, YK, and AHK: edited the final version of the manuscript. All authors reviewed and agreed on the manuscript and ensured that any questions on the work are appropriately resolved.

## Funding

This work was supported by the Healthcare Engineering Innovation Center (HEIC) award: 8474000132 and the Khalifa University award: 8474000174, CIRA 2019-023 awarded to AHK.

## Conflict of interest

The authors declare that the research was conducted in the absence of any commercial or financial relationships that could be construed as a potential conflict of interest.

## Publisher's note

All claims expressed in this article are solely those of the authors and do not necessarily represent those of their affiliated organizations, or those of the publisher, the editors and the reviewers. Any product that may be evaluated in this article, or claim that may be made by its manufacturer, is not guaranteed or endorsed by the publisher.
